# Design, Preparation, and Characterization of Novel Calix[4]arene Bioactive Carrier for Antitumor Drug Delivery

**DOI:** 10.3389/fchem.2019.00732

**Published:** 2019-11-07

**Authors:** Lin An, Jia-wei Wang, Jia-dong Liu, Zi-ming Zhao, Yuan-jian Song

**Affiliations:** ^1^College of Pharmacy, Xuzhou Medical University, Xuzhou, China; ^2^Jiangsu Key Laboratory of New Drug Research and Clinical Pharmacy, Xuzhou Medical University, Xuzhou, China; ^3^Department of Genetics, Research Facility Center for Morphology, Xuzhou Medical University, Xuzhou, China

**Keywords:** amphiphilic calixarene, bioactive carrier, drug delivery, self-assembly, doxorubicin

## Abstract

An amphiphilic and bioactive calix[4]arene derivative **8 (CA)** is designed and successfully synthesized from *tert*-butyl calix[4] arene **1** by sequential inverse F-C alkylation, nitration, O-alkylation, esterification, aminolysis, reduction, and acylation reaction. The blank micelles of **FA-CA** and doxorubicin (DOX) loaded micelles **FA-CA-DOX** are prepared subsequently undergoing self-assembly and dialysis of **CA** and DSPE-PEG_2000_-FA. The drug release kinetics curve of the encapsulated-DOX micelle demonstrates a rapid release under mild conditions, indicating the good pH-responsive ability. Furthermore, the cytotoxicity of DOX-loaded micelle respect to the blank micelle against seven different human carcinoma (A549, HeLa, HepG2, HCT116, MCF-7, MDA-MB231, and SW480) cells has been also investigated. The results confirm the more significant inhibitory effect of DOX-loaded micelle than those of DOX and the blank micelles. The CDI calculations show a synergistic effect between blank micelles and DOX in inducing tumor cell death. In conclusion, **FA-CA** micelles reported in this work was a promising drug delivery vehicle for tumor targeting therapy.

## Introduction

Malignant tumors are one of the most severe diseases currently threatening human health. Clinically, conventional chemotherapy remains the primary means of cancer treatment (Galsky et al., 2016; Gupta et al., 2018) for it can effectively kill cancer cells. Unfortunately, during administration, severe toxic side effects on healthy tissues lead to various complications (Livshits et al., 2014; Makin, 2014). In addition, chemotherapy is subject to problems such as poor solubility, poor long-circulation, low bioavailability and lackness of specificity (Corot et al., 2006; Guo et al., 2013; Qi et al., 2013; Shah et al., 2014), which have limited its clinical applications.

Drug delivery system that can specifically deliver drug has been used as a powerful strategy to alleviate or even address these issues (Hossen et al., 2019). A large number of clinical trial results have successful approved that the ideal drug carriers can comprehensively overcome various biological obstacles during system administration and achieve optimal anti-tumor effect (Whitehead et al., 2009; Gomes-da-Silva et al., 2012; Zhang et al., 2012): (a) Blood barrier: the drug carrier need to maintain a long cycle time in the blood circulation; (b) Tumor targeting: specific enrichment in tumor tissue; (c) Tumor tissue barrier: penetration into various parts of the tumor; (d) Cell membrane barrier: effective entry into tumor cells; (e) Intracellular barrier: rapid release of the drug to the target site.

Supramolecular chemistry, predominantly based on the intermolecular (host-guest, hydrogen bonding, π-π stacking, charge-transfer) interactions can solve effectively some limitations impeding traditional chemotherapy (Murray et al., 2017; Zhou et al., 2017). By taking advantage of supramolecular drug delivery system, the loading or release of drugs can be controlled precisely Drug carrier system based on supramolecules has gradually become an emerging area of interest. Supramolecular host compounds mainly include cucurbituril (Plumb et al., 2012; Isaacs, 2014; Oun et al., 2014; Assaf and Nau, 2015; Barrow et al., 2015; Chen et al., 2017), Pillararene (Duan et al., 2013; Wheate et al., 2016; Huang et al., 2017; Li et al., 2017; Cheng et al., 2019) cyclodextrin (Lu et al., 2015; Bhunia et al., 2018), crown ether (Kumbhat and Singh, 2018) and calixarene (Narkhede et al., 2018). Calixarenes, which are formed by condensation of multiple phenol units and formaldehyde, are considered to represent the third-generation of host-guest supramolecular chemistry. The natures of the basic moiety, including the flexible and variable cavity, excellent biocompatibility and low cytotoxicity, make them ideal as delivery platform for drugs and other chemical molecules (Basilio et al., 2012; Guo and Liu, 2014; Zhou et al., 2015). As potential drug carriers, calixarenes can accommodate different drugs into the hollow cavity resulting in the formation of host–guest inclusion complex (Fahmy et al., 2017). On the other hand, an extremely different drug delivery method has been provided, in which drugs could be encapsulated into nanoparticles by self-assembly behavior of functional calixarene (Chen et al., 2016).

Very recently, calixarenes and their derivatives have been reported as anticancer agents by smart feasible modification of their basic core and rims (Coleman et al., 2007; Yousaf et al., 2015; Naseer et al., 2017). For example, calix[4]arene dihydrophosphonic acid exhibited an effective antitumor activity on fibrosarcoma, melanoma and leukemic cells (Coleman et al., 2010). Neagu et al. (2010) reported *p*-sulfonatocalix[6]arene and calix[8]arene had the photodynamic activity in human K562 myelogenous leukemia cell line. In addition, calix[4]arene amide derivatives have been revealed to target galectin-1 as allosteric inhibitors (Dings et al., 2012, 2013; Astorgues-Xerri et al., 2014; Paz et al., 2018). Previously, we have synthesized calix[4]arene polyhydroxyamine derivative **CLX-4** (An et al., 2016), in which the N-(2-hydroxyethyl) amide group was introduced into the 26,28-position at the lower rim. The *in vitro* evaluation indicated its obvious inhibitory against human carcinoma A549, SKOV3, SW1990, Hela, Raji, and MDA-MB-231 cells.

**Figure 1:**
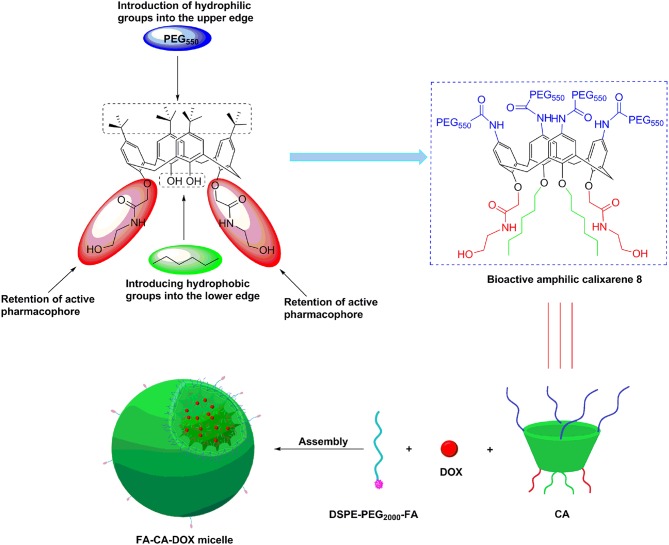
Design of bioactive amphlic calixarene and schematic representation of micelle formation.

Those advantages provide us an exciting new strategy of developing novel bioactive calixarene drug delivery systems, whom can play dual role of drug-loading and synergistic effect. Doxorubicin (DOX) is a commercially used anthracycline anticancer drug, which can be mainly used in the treatment of acute and chronic leukemia, malignant lymphoma. However, the application of DOX was limited due to its poor aqueous solubility, toxicity and acquired drug resistant. In an effort to resolve the limitation of DOX clinically and in continuation of our previous work, herein, we report on a novel DOX delivery system based on bioactive amphlic calix[4]arene polymer micelles (Figure 1).

In our design, the biological activity of the calixarene **8** (**CA**) is guaranteed by retaining the N-(2-hydroxyethyl) amide active groups on 26,28-position at the lower rim; the hydrophobicity is enhanced by introducing long-chain alkyl groups on the 25,27-position; PEG_550_ chain is attached to the upper rim to obtain excellent hydrophilic property; the amphiphilic structure is conducive to the self-assemble formation of micelles; the hydrophobic inside and hydrophilic outside characteristics can make the N-(2-hydroxyethyl) amide group hide in the micelle and hardly to contact with normal cells in the blood circulation, leading to improvement of the safety of carrier. In order to improve the tumor targeting of **CA** micelles, folate derivative (DSPE-PEG_2000_-FA) was combined with **CA** to form FA modified micelles **(FA-CA)**. The doxorubicin (DOX) loaded micelles **FA-CA-DOX** are prepared by the self-assembly of **CA**, DOX with targeting folate (FA) receptor. The calixarene micelles obtained are characterized by dynamic light scattering, transmission electron microscopy techniques in terms of hydrodynamic diameter, ζ-potential and morphology. In terms of cellular uptake, the introduction of FA was in favor of the internalization of **CA** micelles in tumor cells. Blank micelles and DOX-loaded micelles have significant cytotoxic activity in different tumor cell lines. Altogether, These results demonstrate that amphiphilic molecules based on calix[4]arene are capable of dual anti-tumor and drug delivery ability. Nevertheless, pharmacokinetic studies are out of the scope of the present work.

## Materials and Methods

### Synthesis and Characterization

#### Synthesis of Amphiphilic Calixarene Derivative 8

**Figure 2:**
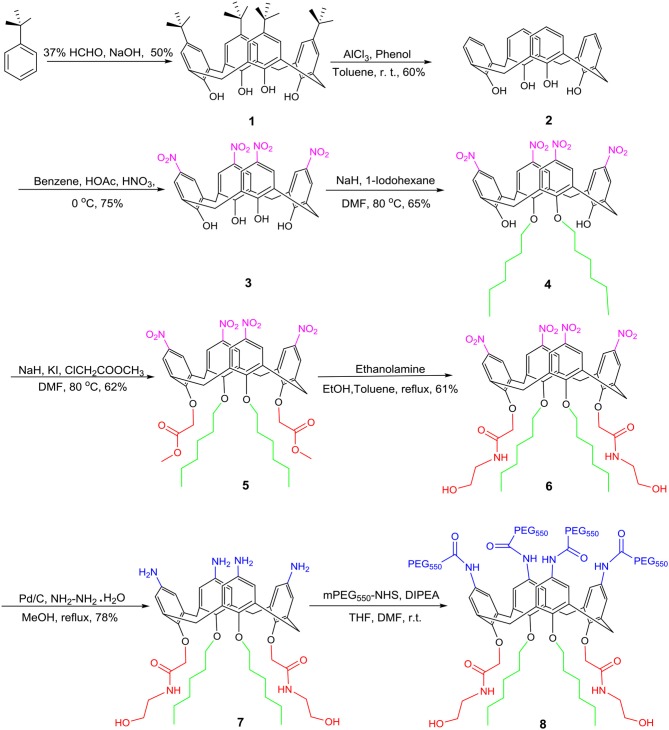
Synthesis of amphiphilic calixarene derivative **8**.

The synthesis of amphiphilic calixarene derivative **8** proceeded as shown in Figure 2. Using *p-tert*-butylphenol as a raw material, compound **1** was obtained by a condensation reaction in a yield of 50% (Gutsche et al., 1986). Subsequently, the *tert*-butyl group of the upper rim was removed by a reverse Friedel-Craft (F-C) reaction to obtain compound **2** in a yield of 60%. The compound **2** was further nitrated using a mixed solution of concentrated nitric acid and glacial acetic acid to afford compound **3** in 75%, which was followed by alkylation with hexyl iodide to give compound **4** in a yield of 65%. In a similar manner, compound **4** subjected to electrophilic substitution with methyl chloroacetate resulted in compound **5** in 62%. Subsequently, aminolysis of compound **5** by ethanolamine to give compound **6** in 61% yield. Compound **6** was reduced in ethanol by Pd/C and hydrazine hydrate to give compound **7** in 78%. At last, the final compound **8** was synthesized by the acylation of compound **7** with NHS-PEG_550_. The compound **8** was characterized and confirmed by ^1^H, ^13^C NMR, and high-resolution mass spectrometry.

#### Hydrodynamic Diameter and ζ-Potential Measurements

The average particle size and ζ-potential of self-assembled micelles were determined using a nanoparticle-Zeta potentiometer (Nicomp 380/ZLS) with the following specifications: sampling time: automatic; number of measurements: 3 per sample; viscosity: 0.933 cP; refractive index: 1.333; scattering angle: 90°; λ = 633 nm; temperature: 25°C. Instrument performance was checked with 90 nm monodisperse latex beads (Coulter) or DTS 50 standard solution (malvern) for DLS before each series of experiments.

#### Transmission Electron Microscopy

TEM micrographs of unloaded (blank) and DOX-loaded calixarene micelles were taken using a transmission electron microscope (Tecnai Spirit G2 TWIN). Sample was prepared according to the following procedure: 0.1 mL of the micelles suspension was placed on the front side of the copper mesh, allowed to stand for 15 min, and the excess suspension was blotted dry with filter paper. 0.1 ml of 4% uranyl acetate negative dye was dropped onto the front side of the copper mesh, stained for 3 min, and then the remaining negative dye solution was blotted with filter paper. The measurements were carried out after naturally drying.

#### Preparation of Blank Micelles

A blank micelle **FA-CA** suspension was prepared by the self-assembly ability of the amphiphilic calixarene derivative **8** (**CA**) using dialysis techniques. Twenty milligrams of **CA** and 2 mg of DSPE-PEG_2000_-FA were dissolved in DMSO and the solution was placed in a dialysis bag (MWCO: 1,000 Da), further dialyzed against 1,000 mL of distilled water for 24 h (change water every 12 h). Collection of dialysate was subjected to filter with a 0.45 μm filter to obtain a blank micelle suspension **FA-CA** stored in a 4°C refrigerator.

#### Preparation of DOX-Loaded Micelles

The drug-loaded micelles were prepared according to the similar procedure described above with the exception of 4 mg of DOX was additionally added to the DMSO solution. After dialysis, the dialysate was filtered through a 0.45 μm filter to remove unembedded DOX and other possible precipitates to give a DOX-loaded micelles suspension (**FA-CA-DOX**). Store in a 4°C refrigerator. The drug-loaded micelles **CA-DOX** containing no folic acid were prepared in the same manner as above but without the addition of DSPE-PEG_2000_-FA.

#### Determination of DOX Loading Capacity

To prepare the samples, the DOX-loaded calixarene micelles were centrifuged (5,000 r/min, 15 min), the supernatant was collected, and the supernatant was freeze-dried to obtain a lyophilized powder. The lyophilized powder was dissolved in methanol, and the absorbance at 490 nm was measured at 25°C using a microplate reader. The content of DOX was calculated from a standard curve. The drug loading content and encapsulation efficiency are calculated by the following formulae:

$$\begin{array}{l} \text{DLC }\left(\%\right)\;=\;\frac{\text{The quality of the encapsulated drug}}{\text{The total mass of drug }-\text{ loaded micelles}}\times 100\%\\ \text{ EE }\left(\%\right)=\;\frac{\text{The quality of the encapsulated drug}}{\text{the total drug used in the formulation}}\times \;100\% \end{array}$$

#### *In vitro* DOX Release Kinetics

*In vitro* release of DOX from **FA-CA-DOX** micelles was studied using methods similar to those reported in previous studies (Lu et al., 2011). Briefly, *in vitro* DOX release kinetics studies were performed by dialysis (MWCO: 10 kDa) in phosphate buffered saline (PBS, 0.01 M, pH 7.3/6.5, 3% SDS) at various pH conditions. The prepared DOX-loaded micelles suspension was dialyzed against 5 mL of PBS under constant agitation. All of the extra dialysis solutions was taken at the scheduled sampling time, measured by high- performance liquid chromatography (HPLC) at 254 nm, and supplemented with 5 mL of pure water as an extradialysis solution. The cumulative release of DOX was calculated from the standard curve. HPLC parameters: column: Agilent TC-C18 (4.6 × 250 mm, 5 μm), detection wavelength: 254 nm, mobile phase: acetonitrile: water (1:1), flow rate: 1 mL/min, injection volume 20 μL, The column temperature was 37°C.

### Cell Culture

The tumor (A549, HeLa, HepG2, HCT116, MCF-7, MDA-MB231, SW480) cell lines used in this study were kindly provided from the cell bank of the Chinese Academy of Sciences. The cells were grown in DMEM cell culture medium containing 1% penicillin-streptomycin solution. The cells were stored at 37°C in a saturated humidity environment of 5% CO_2_.

### *In vitro* Uptake Assay

#### Fluorescence Microscopy Assay

Fluorescence microscopy assay was performed by observing the uptake of DOX micelles by tumor cells. In contrast to the conventional DOX-loaded micelle preparation, in this experiment, 1 mg of the fluorescent substance DSPE-PEG-FITC was previously added to 2 mL of DMSO to form a micelle together with **CA**, and the position of the micelles was marked by green fluorescence of FITC. Briefly, after the drug-loaded micelles were incubated with HeLa cells for 4 h, the cells were washed three times with 0.01 M PBS buffer at pH 7.4. Subsequently, the cells were fixed with 4% paraformaldehyde for 10 min, and then the cells were washed three times with 0.01 M PBS buffer at pH 7.4. Next, the nuclei were stained with DAPI dye for 5 min in the dark, the stain was removed, and the cells were washed three times with 0.01 M PBS buffer, pH 7.4, and stored with 0.9% NaCl solution. At the time of the test, the coverslip containing the cells were placed on a glass slide, observed under an inverted fluorescence microscope, and taken the fluorescent photograph.

#### Mechanism of Micelle Uptake

The HeLa cells were pre-incubated with 100 μL of different uptake inhibitor solution for 2 h at 37°C, 5% CO_2_, followed by half of the medium containing the inhibitor was removed. The DOX-loaded micelles suspension was added and incubated for 4 h, and then, the supernatant was removed, the cells were lysed with RIPA lysate. After treatment, the absorbance was measured at a wavelength of 490 nm on a 96-well plate reader, and the difference in drug uptake of each group was compared.

### Cytotoxicity Study

Cytotoxicity tests were performed on free DOX, precursor compound **7**, blank micelles, and DOX-loaded micelles by SRB kit (KeyGEN BioTECH). Briefly, tumor cells were incubated with different preparations for 72 h (free DOX concentrations ranging from 0.03 to 10 μM, for compound **7** ranging from 0.075 to 25 μM, **CA** concentrations in blank micelles ranging from 0.075 to 25 μM, and DOX-loaded micelles with DOX concentrations ranging from 0.03 to 10 μM). After treatment according to the kit instructions, the absorbance values were measured at 515 nm using a microplate reader.

### Statistical Analysis

Data were calculated using the non-parametric variance Comparisons among groups were statistically analyzed. *P* < 0.05 was considered statistically significant. Statistical analyses were conducted using SPSS 16.0 (SPSS, Chicago, IL, USA).

## Results

### Preparation and Characterization of Amphiphilic Calixarene Derivative 8 and Its Micelles

**Figure 3:**
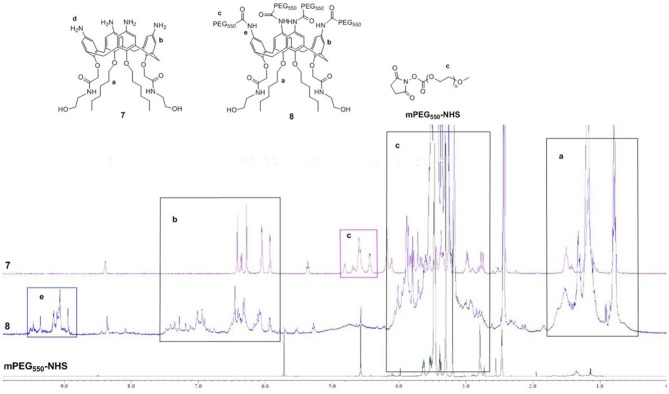
^1^H NMR spectrum comparison of calixarene derivative **7**, **8**, and mPEG_550_-NHS.

According to the previous research results of our group (An et al., 2016), amphiphilic calixarene **8** was synthesized by a series of derivatizations using *p*-*tert*-butylphenol as raw material. The representative products in the reaction were characterized by ^1^H NMR, ^13^C NMR and high-resolution mass spectrometry (see Supplementary Material). ^1^H NMR spectrum comparison of calixarene derivative **7**, **8** and mPEG_550_-NHS was shown in Figure 3.

As seen in Figure 3, the green line at the bottom represents ^1^H NMR spectra of mPEG_550_-NHS, whereas the purple and blue areas represent those of precursor **7**, target compound **8**, respectively. Besides, five main regions of **a**, **b**, **c**, **d** and **e** are marked out for the comparison. The similar upfield multiplets displaying in the region a represented hexyl groups, which cannot be distinguished in the precursor **7** and compound **8**. As illustrated in the area **b**, precursor **7** shows distinct signals for the aromatic protons which appears as a set of at 6.44 ppm (s), 6.38 ppm (d), 6.31 ppm (s), 6.08 ppm (d), and 5.96 ppm (d), whereas the chemical environment of aromatic protons in compound **8** has changed slightly leading to the appearance of broad multiplets at 7.30–6.07 ppm due to the introduction of mPEG_550_-NHS group. Thus, the minor difference is observed. Just for that reason, the chemical shifts in region **c** of compound **8** increase significantly and appear as broad multiplets ranging from 4.10 to 3.00 ppm, corresponding to the -CH_2_CH_2_O-, Ar-CH_2_-Ar and-CONHCH_2_CH_2_- groups. Most importantly, the signal of PEG_550−_CONH-Ar group in compound **8** appears at 9.00 ppm, which can be used as an characteristic feature of the presence of PEG chain. Therefore, we have successfully synthesized the target compound **8**.

**Figure 4:**
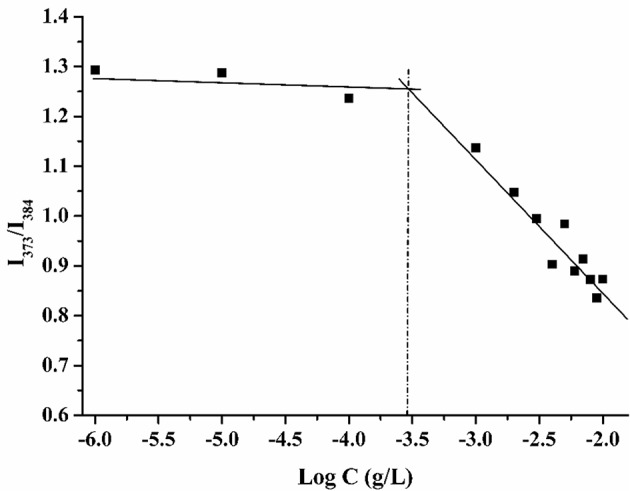
Plots of intensity ratio (I_373_/I_384_) from fluorescence emission spectra of pyrene vs. log C of calixarene derivative **8**.

The critical micelle concentration of compound **8** was determined by the fluorene probe method. The fluorescence spectrum of pyrene in water shows five major peaks, and the intensity ratio of the first peak (I_1_ at 373 nm) to the third peak (I_3_ at 384 nm) can reflect the polarity of the microenvironment in which the probe is located (Goddard et al., 1985). When the micelles formed, the ratio of I_373_ to I_384_ suddenly changed. As shown in Figure 4, the critical micelle concentration of compound **8** is 0.25 mg/L.

DOX is a common hydrophobic antitumor drug. It can be accumulated in the hydrophobic layer of micelles and liposomes, thereby improving its water solubility and toxic side effects (Liang et al., 2018; Zhang et al., 2019). In this experiment, calixarene bioactive micelles **FA-CA** were prepared according to the dialysis method (Takata et al., 2017). A DMSO solution containing calixarene derivative **8** and other excipients was placed in a dialysis bag and dialyzed against distilled water to obtain a micelle suspension. DOX-loaded micelles **FA-CA-DOX** were prepared similarly with a CA: DOX molar ratio of 5:1. DOX is concentrated in the hydrophobic core inside the micelle. The drug loading content, encapsulation efficiency, as well as the particle size and zeta potential of micelles are shown in Table 1.

**Table 1:** Particle size and zeta potential of **FA-CA** and **FA-CA-DOX** micelles.

**Micelle**	**Mean diameter(nm)**	**PDI^**a**^**	**DLC^**b**^ (wt%)**	**EE^**b**^ (wt%)**	**ζ-potential (mV)**
**FA-CA**	75.1 ± 2.0	0.42 ± 0.02	–	–	0.96 ± 0.67
**FA-CA-DOX**	81.8 ± 3.6	0.36 ± 0.02	6.85 ± 0.6	41.10 ± 3.60	0.63 ± 0.25

a *Polydispersity index of blank micelles/DOX-loaded micelles obtained from DLS measurements*.

b *Measured by a UV spectrophotometer at 490 nm; “–” Indicates that the drug is not contained*.

*Data show mean ± SD of three independent determinations*.

As shown in Table 1, the DOX loading efficiency and encapsulation efficiency of prepared calixarene micelles are 6.85 ± 0.6% and 41.10 ± 3.60% (W/W, *n* = 3), respectively. The drug loading content is in the rational level. The PDIs are 0.96 ± 0.67 and 0.63 ± 0.25. The ζ-potential tends to be 0 mV similar to non-ionic surfactants, but it still maintains good stability due to its steric repulsion. The blank micelles have particle size of 75.1 ± 2.0 nm, whereas that of drug-loaded micelles is higher (81.8 ± 3.6 nm). The slightly increase in particle size described is based on the data of particle size measured by DLC. It is in consistent with the literature reported (Liu et al., 2007), mainly due to the expansion of the core and shell of micelles caused by the crowding of DOX molecules in the hydrophobic cavity of micelles.

**Figure 5:**
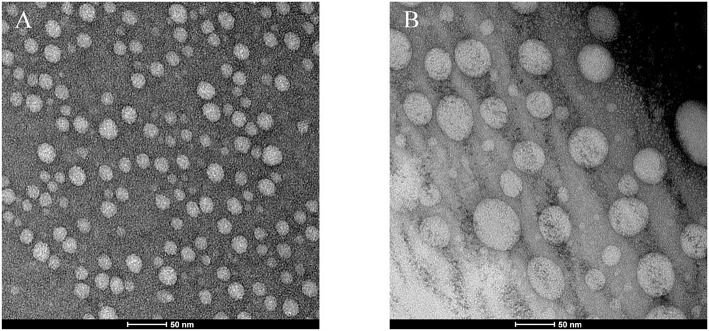
TEM images of blank micelles (**FA-CA**) **(A)** and DOX-loaded micelles (**FA-CA-DOX**) **(B)**.

The transmission electron micrographs are exhibited in Figure 5. As observed in TEM images (Figure 5), micelles exhibited a quasi-spherical shape and were moderately uniform in size distribution. In particular, the particle size of blank carriers appears to be smaller than that of DOX-loaded carriers. The behavior maybe rationalized speculated that the dry particles collapse on the surface of micelles for the drying process. The particle size becomes smaller, and especially the collapse of blank carriers is more obvious. Therefore, the particle size of blank carriers appears to be about 50% smaller than that of drug carriers. In addition, the particle size estimated by TEM was smaller that of particle measured with DLS because that DLS measures the hydrodynamic diameter of the particles core along with the solvation layer attached to the particles (Zhao et al., 2015).

**Figure 6:**
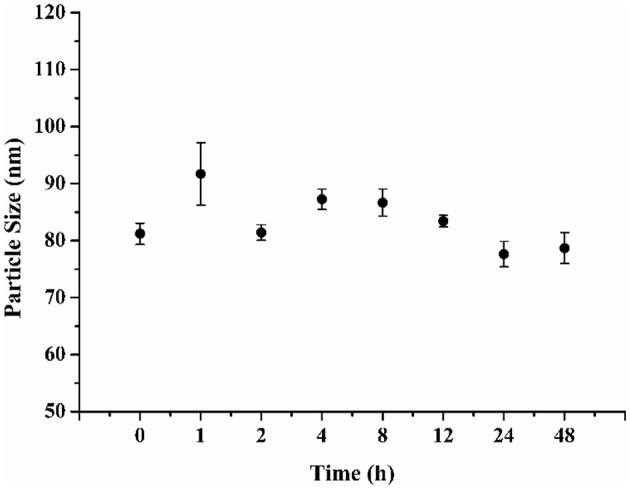
Changes in particle size of **FA-CA-DOX** micelles in PBS (0.15 M, pH 7.4) at 4°C.

When the **FA-CA-DOX** micelles were stored at 4°C during 48 h, they remained stable and no obvious change in particle size was observed (Figure 6). The results suggested that **FA-CA-DOX** micelles would provide good stability.

**Figure 7:**
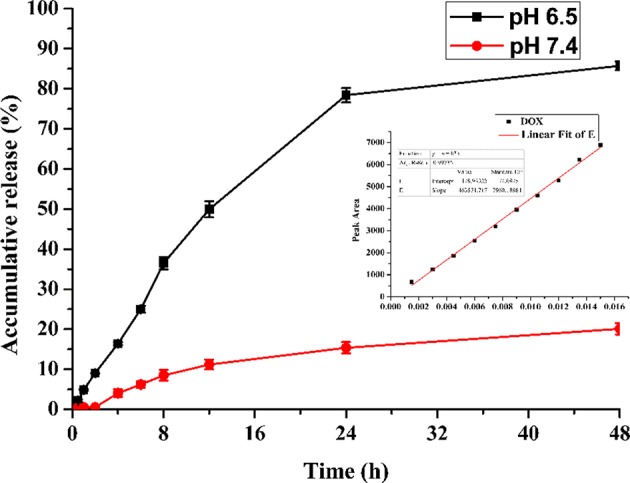
Accumulated DOX-release profiles of **FA-CA-DOX** micelles in 0.15 mol/L PBS (pH 6.5, pH 7.4) containing 3% SDS. Inset: linear curve of DOX.

The DOX release kinetics of **FA-CA-DOX** micelles conforms to a hyperbolic profile with an initial burst within 10 h, followed by a sustained delivery over 24–48 h (Figure 7). It is speculated that the fast release of DOX comes from the part of the micelle near the interface between the hydrophilic layer and the hydrophobic layer. The slow-releasing portion accounts for the DOX encapsulated in the core of the micelle. As the drug spreads continuously, the release rate is correspondingly reduced.

It is noted that the release profile of DOX indicates **FA-CA-DOX** is pH-responsive, and the release rate under slightly acidic conditions is much greater than normal physiological conditions (pH 7.4). This is mostly related to the amido linkage (-CONH-PEG_550_ group) at the upper rim of the aromatic rings in the carrier, which is generally thought to be pH-sensitive (Cao et al., 2014; Li et al., 2018; Panja et al., 2018). The drug release process itself is a passive diffusion behavior: DOX loaded in micelles is released into the medium through passive diffusion, amido linkage is partially hydrolyzed under weak acidic conditions, which can destroy the integrity of the micelle structure and moreover make the DOX loaded in the micelles release faster. In a contrast, under neutral conditions, the integrity of micelles is maintained well, and the release rate of DOX from micelles to media is much slower. Therefore, most of the released DOX are around the hydrophilic-esterophilic interface of micelles, the DOX containing in the core of micelles is basically not released.

### Cellular Uptake of the Micelle

**Figure 8:**
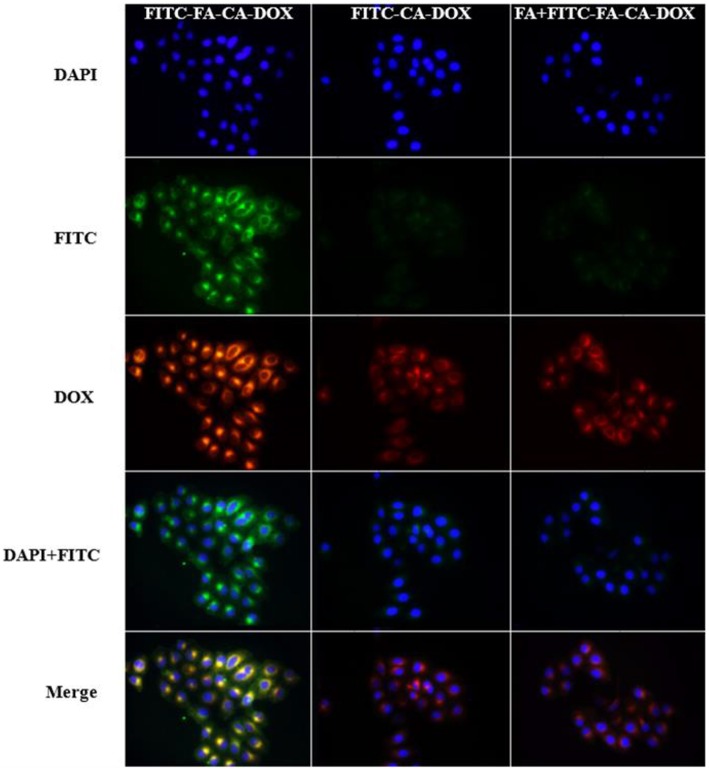
Inverted fluorescence microscope observation of intracellular internalization profiles of **FITC-FA-CA-DOX**, **FITC-CA-DOX**, and free **FA** **+** **FITC-FA-CA-DOX** micelles after incubation with HeLa cells. Blue fluorescence represents DAPI, green fluorescence represents FITC and red fluorescence represents DOX.

HeLa cells are tumor cells that overexpress folate receptors. The uptake of micelles by tumor cells was investigated by incubating DOX-loaded micelles with HeLa cells for 4 h. The micelles have been previously labeled with FITC. As shown in Figure 8, the green fluorescence represents the position of the micelle, which is from the FITC. The red fluorescence is created by DOX itself and represents the position of the drug. Meanwhile, the blue fluorescence is resulted from DAPI and embodies the position of the nucleus. The results ascertained evidence that **FITC-FA-CA-DOX** group presented the best ingestion activity after co-incubation with HeLa cells for 4 h. After that, only a small amount of DOX-loaded micelles were observed to enter the cells. With addition of free FA as a competitive antagonist, the effect of **FITC-FA-CA-DOX** micelles entering the cells was significantly reduced, indicating that FA has a significant positive effect on the micelles entering the cells.

**Figure 9:**
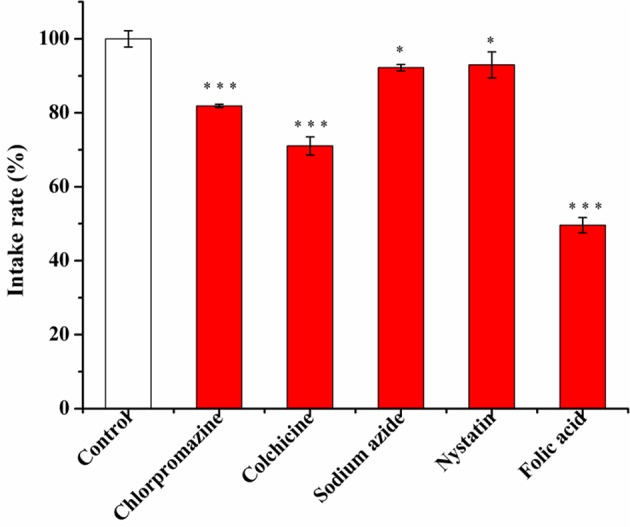
The effect of different inhibitors (chlorpromazine 10 μg/mL, colchicine 50 μg/mL, sodium azide 1 mg/mL, nystatin 6 μg/mL, folic acid 10 μg/mL) on the uptake of **FA-CA-DOX** micelles in cells within 4 h after incubation with cells for 2 h. Data presented as mean ± SD, *n* = 3, ^*^P < 0.05 vs. control group, ^***^P < 0.001 vs. control group.

The profile of cell uptake on micelles after addition of various cellular uptake inhibitors is shown in Figure 9.

As shown in Figure 9, tubulin and clathrin have a great influence on cell uptake. After using the corresponding inhibitors, the cell uptake rate of micelles decreased by 28.96 and 18.14%, respectively.

### Cytotoxic Action of Blank Micelles and DOX-Loaded Micelles

To study the activity of blank micelles **FA-CA** *in vivo*, cytotoxic effect on different seven types of tumor cell cells was evaluated by MTT method. Precursor **7** was added as a control group to verify whether the introduction of hydrophilic PEG chain can affect its activity.

**Figure 10:**
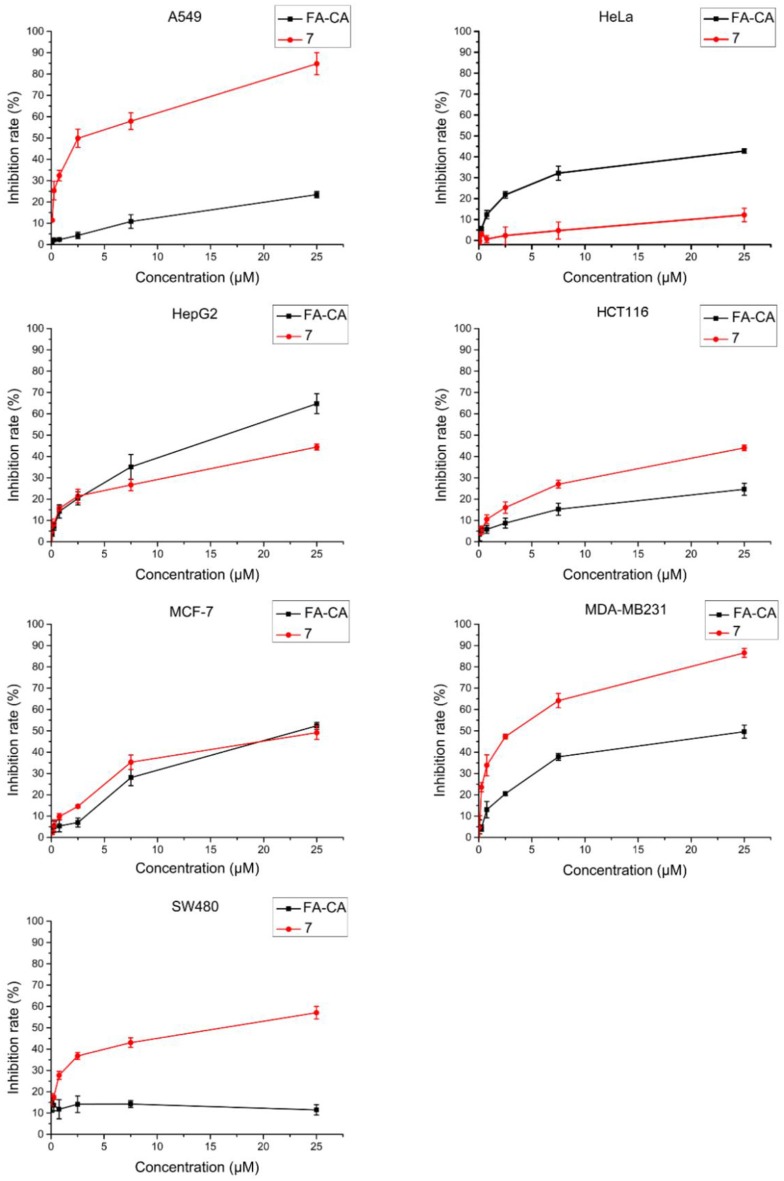
Cytotoxicity of **FA-CA** micelles and compound 7 to different tumor cells at different concentrations measured by the standard SRB assay. Data presented as mean ± SD, *n* = 3.

**Figure 11:**
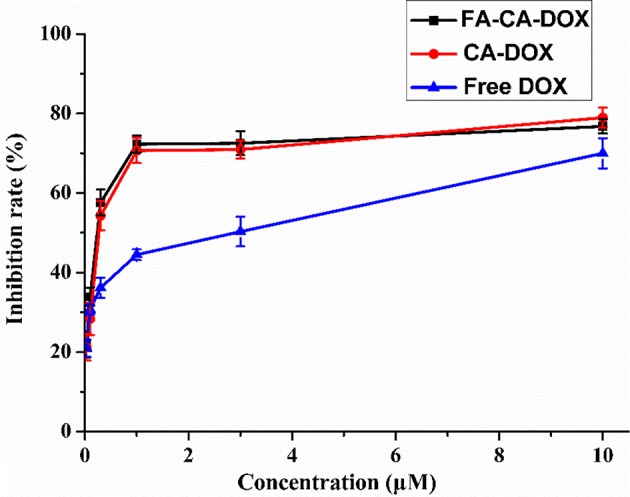
Cytotoxicity of **FA-CA-DOX** micelles, CA-DOX micelles, and free DOX to HepG2 cells at different concentrations measured by the standard SRB assay. Data presented as mean ± SD, *n* = 3.

In this experiment, most of selected tumor cell lines, such as A549, HCT116, MDA-MB231, and SW480 cells were sensitive to DOX. Cell inhibition was assessed 72 h after treatment and dose response curves are shown in Figure 10. The blank micelles **FA-CA** demonstrated much impaired cytotoxic effects on tested A549, HCT16, SW480, and MDA-MB231 cells in comparation with precursor **7**. It was suggested that the introduction of four PEG_550_ chains at the upper rim of blank micelles **FA-CA** probably was to the disadvantage of the cytotoxicity. The reason maybe complicated, which could be explained by the assumption that the increasement of the hydrophilicity and molecular weight attributed to PEG_550_ chains make it difficult to permeate the cell membrane. Besides, the increased steric hindrance, reduced hydrogen bonding caused by PEG chain also lead to the poor pharmacological activity. Interestingly, the blank micelles **FA-CA** was most efficient on the inhibition of HepG2 cells, we performed further cytotoxicity of free DOX, **FA-CA-DOX** micelles and **CA-DOX** micelles against HepG2 cells and the results are shown in Figure 11.

To our satisfaction, the drug-loaded micelles was significantly higher than that of the free DOX, and the IC_50_ value was reduced from 1.52 to 0.31 μM in terms of DOX dose. Compared with **CA-DOX**, the introduction of folic acid in **FA-CA-DOX** did not exert a significant effect on the toxicity. This may be related to the incubation time. After 72 h, the folate-promoting cells can fully enter the cells regardless of the uptake of DOX-loaded micelles. The interactions between blank micelles and DOX are shown in Table 2. In terms of growth inhibition of HepG2 cells, a weak synergy was exhibited at low concentrations (0.3–1.0 μM), CDI < 1.

**Table 2:** Interaction between blank vector and DOX.

**Concentration (μM)**	**DOX**	**10**	**3**	**1**	**0.3**	**0.1**	**0.03**
	**CA**	147	44.1	14.7	4.4	1.5	0.44
**CDI**^**a**^		3.51	1.74	0.98	0.91	1.13	1.08

a *CDI = AB/(A + B), Among them, A and B are the cell activities when each drug is used alone, AB is the cell activity when the two drugs are combined. When CDI < 1, the two drugs have a synergistic effect, CDI = 1, the two drugs have a superposition effect, when CDI > 1, both drugs have antagonistic effects*.

**Figure 12:**
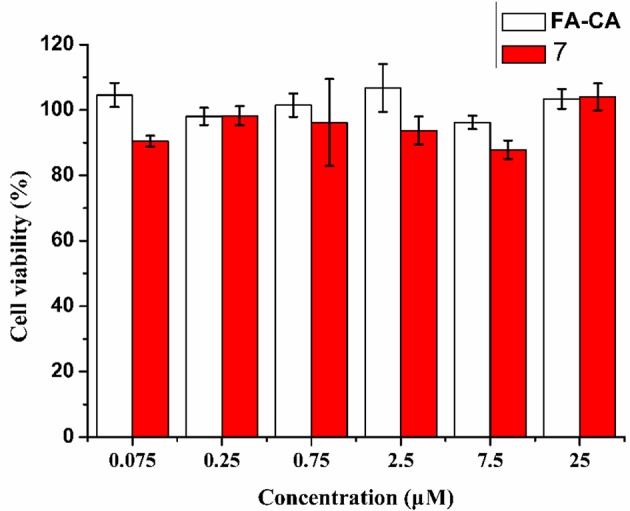
Cytotoxicity of **FA-CA** micelles as well as Compound 7 to HUVEC cell at different concentrations measured by the standard SRB assay. Data presented as mean ± SD, *n* = 3.

To further confirm the safty of the blank micelles **FA-CA** in contrast to the compound **7**, the toxity on normal HUVEC (human umbilical vein endothelial) cells was also investigated. The results shown in Figure 12 proved that our synthesized bioactive calixarene drug is safe because it has no significant toxicity to normal cells at a concentration of 25 μM.

## Discussion

Based on the previous research of our group (An et al., 2016), in the present work, a bioactive drug-loaded calix[4]arene micelle was hoped to construct in an effort to explore its drug encapsulation, delivery and release ability and evaluate its *in vitro* efficacy, especially to reveal the synergy effects. The bioactive calixarene derivative **8 (CA)** is firstly designed and obtained, in which the hydroxylamine group at the lower rim acts as an antitumor active group, the hexyl chain and the benzene ring act as hydrophobic moieties, and the PEG chain at the upper rim acts as a hydrophilic moiety (seen in Figure 1). Due to the cone conformation of the calix[4]arene, the compact compaction of the hydrophobic portion at its narrower rim enhances its self-assembly ability in an aqueous environment (Gallego-Yerga et al., 2015). In the resulting micelles, the hydrophobic tail and anti-tumor active groups are located at the core of the micelle and are expected to be well-suited to contain hydrophobic drugs. It is worth mentioning that, considering the active targeting problem, we introduced folic acid molecules during micelle preparation (Stella et al., 2000). Although the folic acid molecule is not directly covalently modified on the calixarene molecule, it can still achieve the purpose of promoting cellular uptake (Figure 8). Novel DOX-loaded amphiphilic calix[4]arene derivative **8** was successfully synthesized and characterized.

When preparing drug-loaded micelles, DOX is co-dissolved with calixarene derivative **8**, DSPE-PEG_2000_-FA prior to dialysis, allowing DOX to be efficiently integrated into the micelles. The drug-loaded micelles obtained by this method have the drug loading efficiency and encapsulation efficiency were 6.85 ± 0.6 and 41.10 ± 3.60%, which are consistent with the general drug-loading capacity of the nanocarriers (Zhao et al., 2015; Conte et al., 2016). The drug loading has little effect on the properties of the micelles. After the incorporation of DOX, the particle size of the micelles increased only slightly (Table 1). It is confirmed that both the blank micelles and the drug-loaded micelles are quasi-spherical. The particle size of the micelles increased slightly after drug loading due to the drug filling. The possible cause is that the degree of aggregation of the hydrophobic portion of the calixarene is slightly reduced due to the entry of the drug into the hydrophobic core of the micelle (Callari et al., 2017). This can also be reflected in the TEM photo (Figure 5). The particle size measured by DLS is slightly larger than that of TEM. Considering the different principles of the two techniques, this seems reasonable. It is well-known that DLS measures the hydrodynamic diameter of a particle core and a solvation layer attached to the particle (Palma et al., 2007). When the calix[4]arene micelles are dispersed in water, a hydrate layer was formed outside the nanoparticle. However, after sample preparation, the hydration layer was not present under TEM (Pyrz and Buttrey, 2008). In addition, the ζ-potential is still around 0 mV before and after drug loading.

Drug release from drug-loaded micelles is a complex process that can be affected by many factors, including degradation and erosion of the micelles matrix, diffusion of the drug, and binding affinity between the micelle and the drug (Mohanraj and Chen, 2006). The DOX release kinetics from **FA-CA-DOX** micelles are compatible with the assembled micelle structure. Since the drug close to the hydrophilic layer can be rapidly released in the aqueous solution, an initial rapid release period is observed in the release profile. The remaining drug is slowly released by diffusion, which is also a possible cause of sustained drug release. And as the amount of drug remaining inside the micelles decreases, the release rate of DOX becomes slower and slower. It is worth noted that **FA-CA-DOX** micelles also exert a certain pH response ability (Figure 7), which can be explained by partial hydrolysis of the amide bond on the PEG side chain under weakly acidic conditions (Li et al., 2018; Panja et al., 2018). Due to the mild acid sensitivity, partial amide bond breaks under weakly acidic conditions, resulting in the destruction of the hydrophilicity and lipophilic balance of the calixarene molecule, thereby destroying the composition of the micelles, the drug inside the micelles is quickly released.

In this work, the prepared **FA-CA-DOX** micelles were stable for more than 2 days at 4°C and pH 7.4. There is no change in the particle size of the micelles were observed (Figure 6).

At present, the main disadvantage of DOX as an anti-tumor drug is poor water solubility and strong side effects (Zhang et al., 2018). Although DOX·HCl has been developed to improve the water solubility of DOX, it still cannot solve the drawback of side effects (Pu et al., 2013). The calixarene-based bioactive carrier studied here can effectively entrap DOX (Table 1), dissolve it in water without co-solvent, and complete release in the tumor tissue microenvironment (pH 6.5) in about 48 h (Figure 7). In addition, **FA-CA-DOX** micelles are targeted based on the EPR effect of nanocarriers and the molecular targeting effect of folic acid-folate interactions. It can be enriched in the tumor site, reducing the side effects caused by the non-specific distribution of the drug.

According to our previous studies, the IC_50_ value of calix[4]arene derivatives to induce cell death is approximately 1–10 μM (An et al., 2016). In this study, the anti-tumor activity of blank micelles and compound **7** against various types of tumor cell lines are evaluated (Figure 10). The results showed they had varying degrees of inhibitions. The introduction of PEG side chains seemed to have slight disadvantage for the cell inhibition of calixarene regardless of its perfect hydrophilicity. This can be explained in two ways: (a) Since the amino group of the upper rim is amidated, the number of hydrogen bonds formed between the compound and the receptor on the cell membrane or a related protein is reduced, resulting in a weakening of the strength of the interaction of the compound with the cell. Therefore, the pharmacological activity is lowered; (b) The interaction between a compound and its receptor is closely related to the stereo configuration of the compound. This is especially true for supramolecular compounds. The PEG chain attached to the upper rim greatly would raise the molecular weight, decrease the lipophilicity and cause more steric hindrance, as well as reduce its pharmacological activity. Interestingly, in experiments with HepG2 cells, the inhibition of HepG2 cells was increased after the introduction of the PEG side chain. This suggests that the inhibitory effect of our compounds on tumor cells is closely related to the type of tumor cells. In summary, the blank micelles we designed does have the ability to inhibit tumor cell proliferation.

The tumor suppressive effects of **FA-CA-DOX** and **CA-DOX** micelles were studied in HepG2 cells, and free DOX was used as a positive control. The cytostatic activity of the formulation was found to be significantly stronger than that of free DOX, but the presence of folic acid did not affect the final tumor suppressive effect. This phenomenon may be due to the co-incubation time of up to 72 h, so even without the folate-folate receptor interaction, the micelles can fully enter the cell to exert an inhibitory effect. In addition, we calculated whether there is a synergistic effect between blank micelles and DOX in inhibiting the growth of HepG2 cells based on cytotoxicity data (Acevedo et al., 2014). As shown in Table 2, the blank micelles did have a weak synergistic effect with DOX at low and medium concentrations, indicating that the overall efficacy improvement was not only related to the promotion of DOX entry into cells, but also to the anti-tumor effect of micelles themselves.

The safety of the formulation is also one of the factors that need not be considered. In the cytotoxicity experiments on normal cell lines (HUVEC cells), we found that the blank vector and the precursor compound **7** maintained a cell activity of more than 90% at a concentration of 25 μM (Figure 12). It is indicated that the calixarene micelles have good safety for normal cells. In addition, in order to increase the accumulation of drugs at the tumor site to reduce side effects and improve the efficacy, the strategy of using PEG as a stealth coating is very effective. It not only has hydrophilicity, but also prevents serum protein and micelles from binding, prolongs the circulation time of the carrier in blood vessels, and cooperates with EPR effect and folic acid targeting to improve the accumulation of drugs in tumor sites. In addition, PEG also has excellent safety. These characteristics make us tend to use PEG as a hydrophilic group.

## Conclusion

To the best of our knowledge, the concept of calixarene-based bioactive carriers reported in this paper was first proposed by our group. By introducing functional groups, the dual function of drug-loading and biological activity can be obtained. The resulting calix[4]arene derivative produces the corresponding calixarene micelles by self-assembly. The drug-loading and drug-releasing properties of the micelles are closely related to the molecular structure of the calixarene. In the cytotoxicity test, the drug-loaded micelles have more antitumor activity than the free DOX formulation, overcoming the problem of poor water solubility of DOX. The proposed nanomicelles are further proved to be biocompatible in the safety study. The introduction of PEG gave micelles a low tendency to adsorb proteins. The presence of folic acid also gives it an active targeting capability in addition to passive targeting mediated by EPR effects, and can be used for tumor-specific drug delivery. This allows them to bypass healthy cells and thus reduce the toxic side effects of DOX. We will continue to carry out relevant *in vivo* pharmacokinetic evaluations in our laboratories in future.

## Data Availability Statement

All datasets generated for this study are included in the article/Supplementary Material.

## Author Contributions

LA and ZZ designed the work. JW and JL made contributions to the experiments and collective data. YS and ZZ provided substantial technical support. LA and JW wrote the paper. All authors extensively discussed the results reviewed the manuscript, and approved the final version of the manuscript to be submitted.

### Conflict of Interest

The authors declare that the research was conducted in the absence of any commercial or financial relationships that could be construed as a potential conflict of interest.
