# Supplementary material for: Design, Preparation, and Characterization of Novel Calix[4]arene Bioactive Carrier for Antitumor Drug Delivery
Source: Front Chem. 2019 Nov 7;7:732. doi: 10.3389/fchem.2019.00732 (PMC6855266; doi:10.3389/fchem.2019.00732)
Supplement: Supplementary file 1 [file Data_Sheet_1.pdf]

SUPPORTING INFORMATION

DESIGN, PREPARATION AND CHARACTERIZATION OF NOVEL CALIX[4]ARENE BIOACTIVE CARRIER FOR ANTITUMOR DRUG DELIVERY

Lin An[1, 2*], Jia-wei Wang[+1,2], Jia-dong Liu[1,2], Zi-ming Zhao[*], Yuan-jian Song[3]

[1] College of Pharmacy, Xuzhou Medical University, Xuzhou 221004, P. R. China
[2] JiangsuKey Laboratory of New Drug Research and Clinical Pharmacy, Xuzhou Medical University, Xuzhou 221004, P. R. China
[3] Department of Genetics, Research Facility Center for Morphology, Xuzhou Medical University, Xuzhou 221004, P. R. China

[+] Jia-wei Wang **contributed equally to this work.**

*** Correspondence:**

Dr Lin An, anlinhx@sina.com.cn

1 *p*-tetra-nitro-calix [4]arene

^{1}H NMR: (DMSO-d_6,400 MHz) δ 8.18 (s, 8H), 4.26 (s, 4H), 3.74 (s, 4H); ^{13}C NMR: **(**DMSO-d_6, 100 MHz) δ 162.91, 138.70, 130.04, 125.19, 31.08; HRMS (ESI) Calcd for $C_{28}H_{20}N_4O_{12}$ ($[M+NH_4]^+$): 622.1421, found: 622.1394.

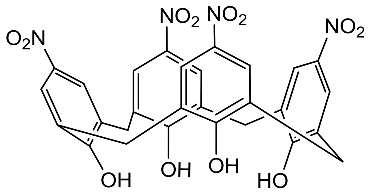

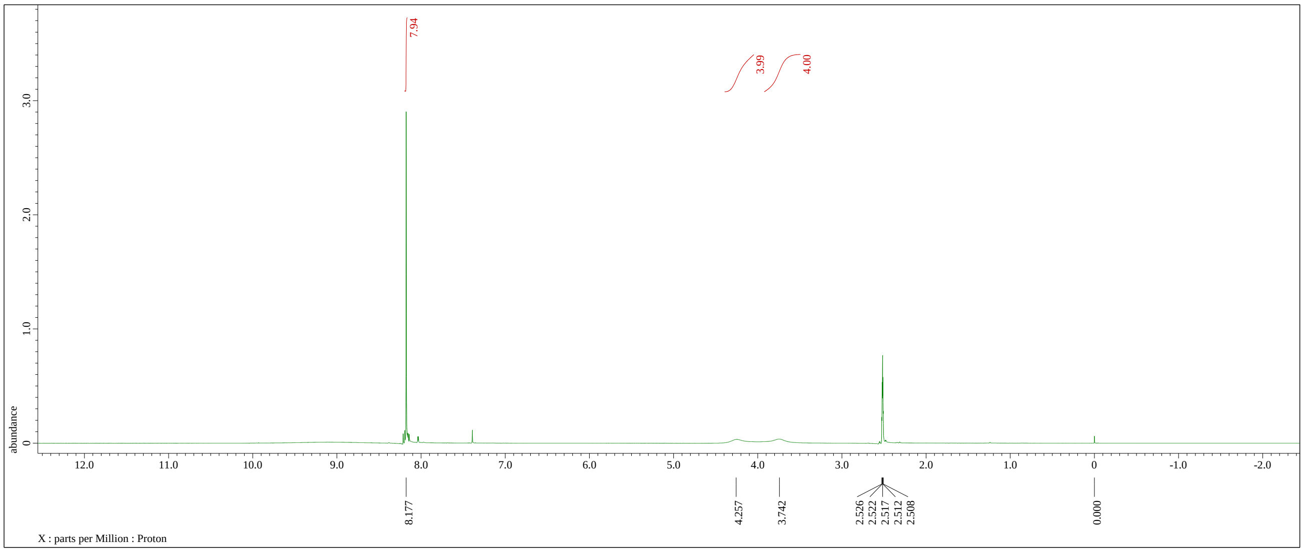

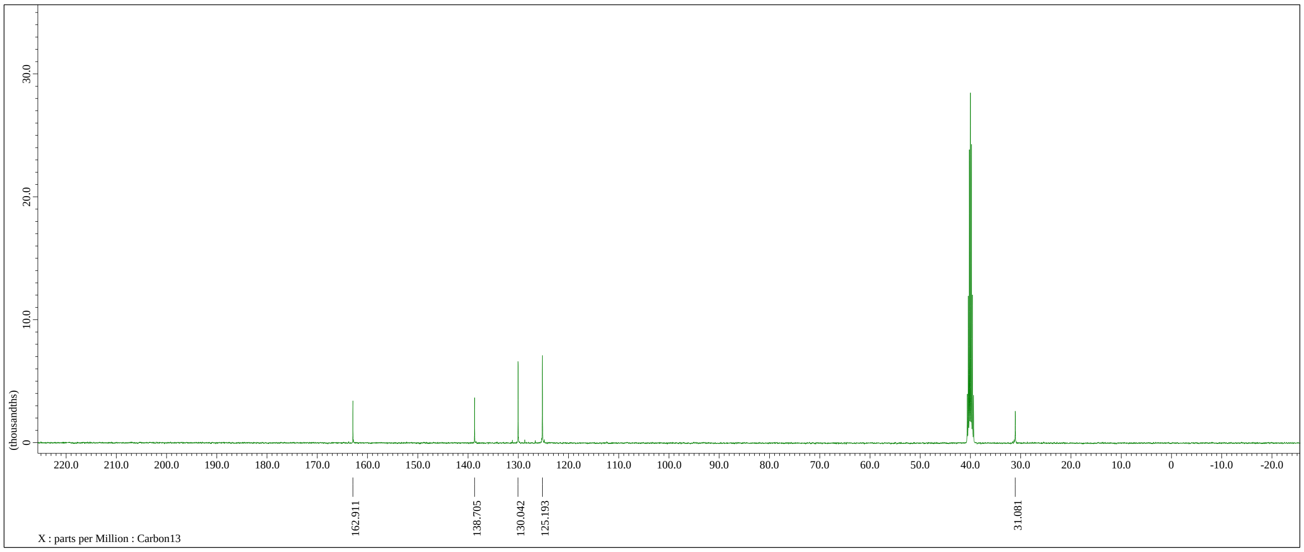

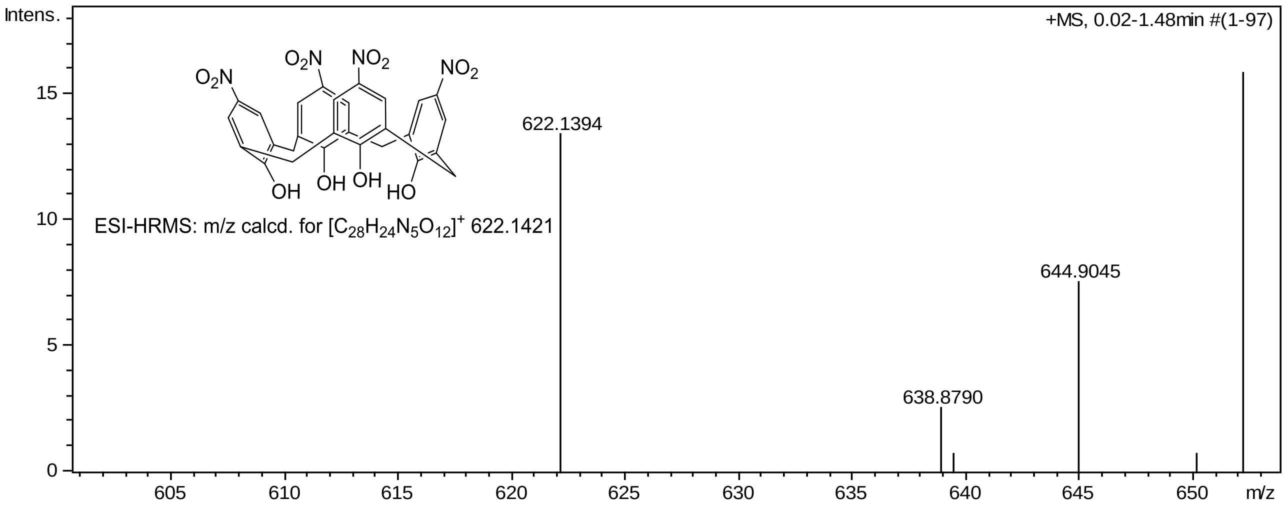

2 5,11,17,23-tetra-nitro-25,27-bis-hexyloxy- calix[4]arene-26,28-diol

^{1}H NMR: ($CDCl_3$, 400 MHz) δ 8.72 (s, 2H), 8.17 (s, 4H), 7.85 (s, 4H), 4.34 (d, *J* = 13.6 Hz, 4H), 4.11 (t, *J* = 6.8 Hz, 4H), 3.65 (d, *J* = 14.0 Hz, 4H), 2.13-2.06 (m, 4H), 1.74-1.66 (m, 4H), 1.51-1.38 (m, 8H), 0.96 (t, *J* = 7.6 Hz, 6H); ^{13}C NMR: ($CDCl_3$, 100 MHz) δ 159.01, 156.91, 145.23, 140.55, 133.43, 126.93, 125.29, 78.26, 31.62, 31.44, 30.01, 25.61, 22.66, 14.13; HRMS (ESI) Calcd for $C_{40}H_{44}N_4O_{12}$ ($[M+Na]^+$): 795.2853, found: 795.2872.

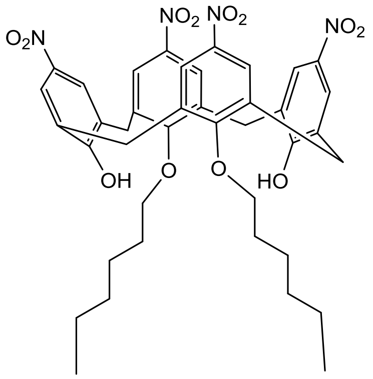

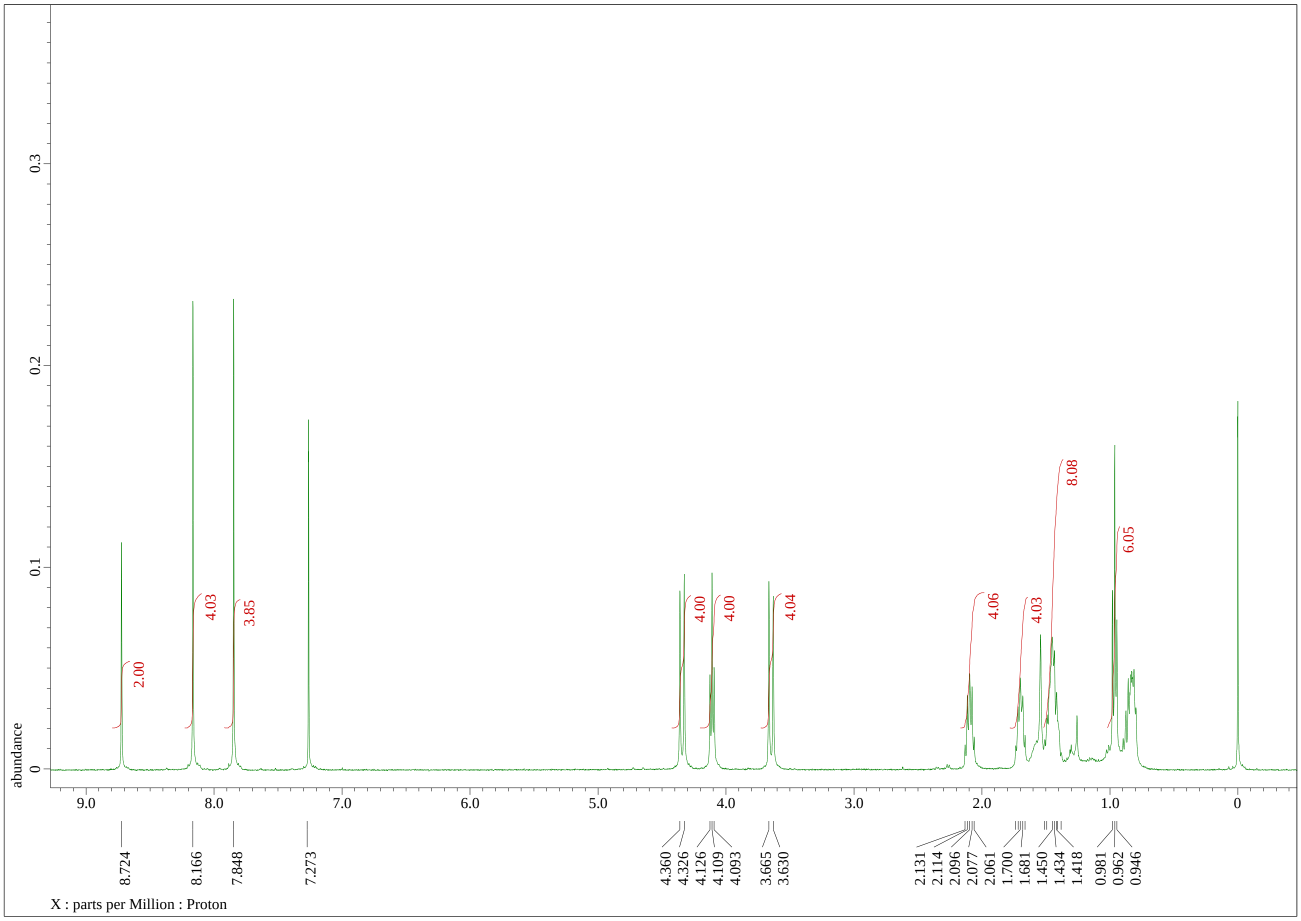
abundance
X : parts per Million : Proton
8.724
8.166
7.848
7.273
4.360
4.326
4.126
4.109
4.093
3.665
3.630
2.131
2.114
2.096
2.077
2.061
1.700
1.681
1.450
1.434
1.418
0.981
0.962
0.946
2.00
4.03
3.85
4.00
4.00
4.04
4.06
4.03
8.08
6.05

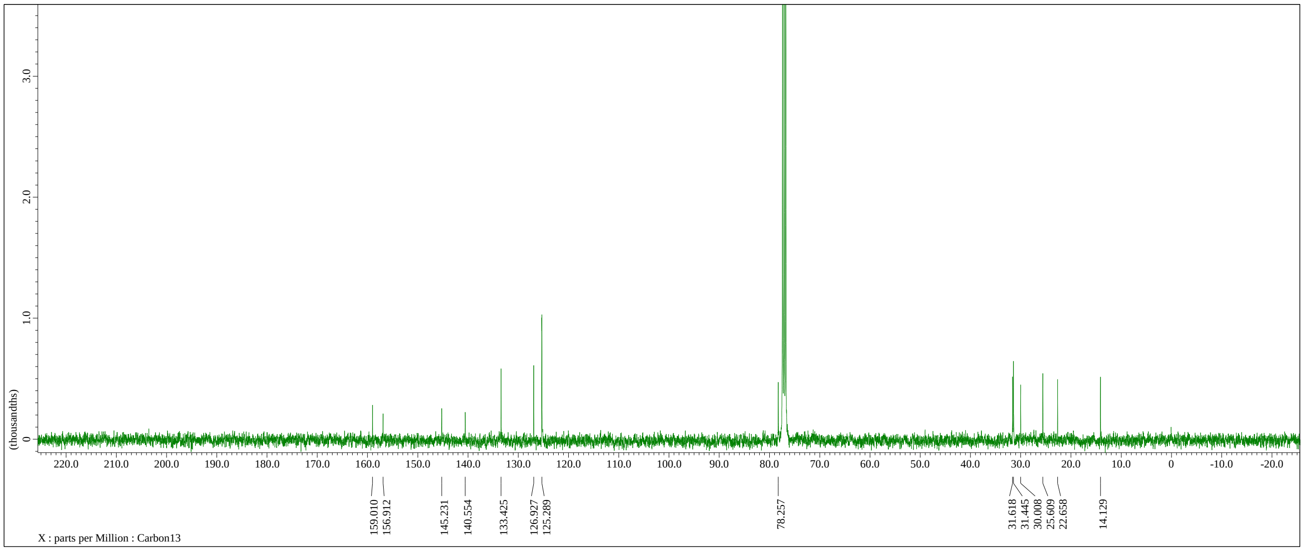
(thousandths)
X : parts per Million : Carbon13
159.010
156.912
145.231
140.554
133.425
126.927
125.289
78.257
31.618
31.445
30.008
25.609
22.658
14.129

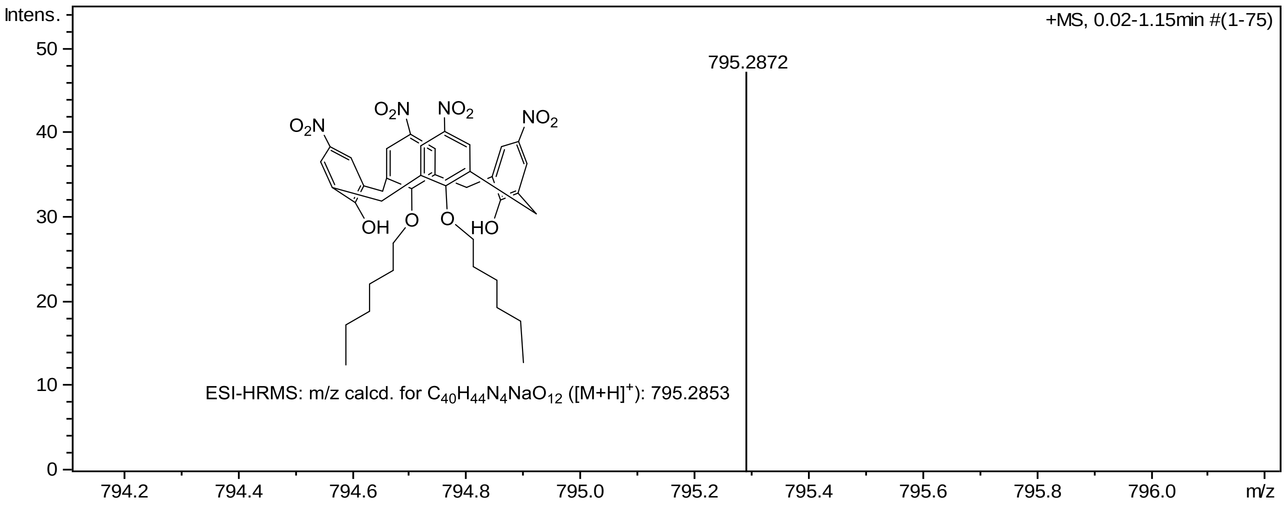

3 5,11,17,23-tetra-nitro-25,27-bis-hexyloxy-26,28-bis-methoxyl-carbonylmethoxyl-calix[4] arene

^{1}H NMR: ($CDCl_3$, 400 MHz) δ 8.27 (s, 2H), 8.11(s, 2H), 7.99 (d, *J* = 2.8 Hz, 2H), 7.13 (d, *J* = 2.4 Hz, 2H), 4.65 (s, 2H), 4.42 (d, *J* = 14.8 Hz, 3H), 4.06 (s, 3H), 3.95 (s, 2H), 3.88 (s, 2H), 3.79-3.77 (m, 2H), 3.75-3.73 (m, 2H), 3.67 (s, 2H), 3.61 (s, 3H), 3.40 (d, *J* = 14.8 Hz, 2H), 1.90-1.97 (m, 4H), 1.49-1.56 (m, 4H), 1.38-1.45 (m, 8H), 0.94 (t, *J* = 7.2 Hz, 6H); ^{13}C NMR: ($CDCl_3$, 100 MHz) δ 169.09, 168.17, 160.89, 160.11, 142.95, 136.29, 134.63, 134.10, 132.20, 126.19, 125.46, 124.83, 124.01, 76.17, 70.97, 67.80, 53.39, 51.76, 34.67, 31.81, 30.48, 25.94, 22.62, 14.11; HRMS (ESI) Calcd for $C_{46}H_{52}N_4O_{16}$ ([M+Na]$^+$): 939.3276, found: 939.3301.

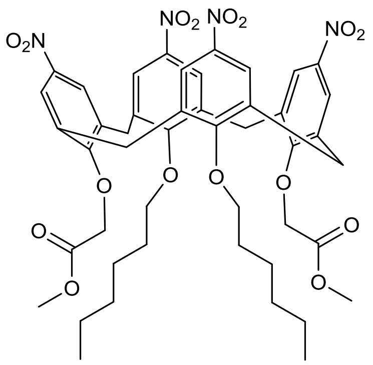

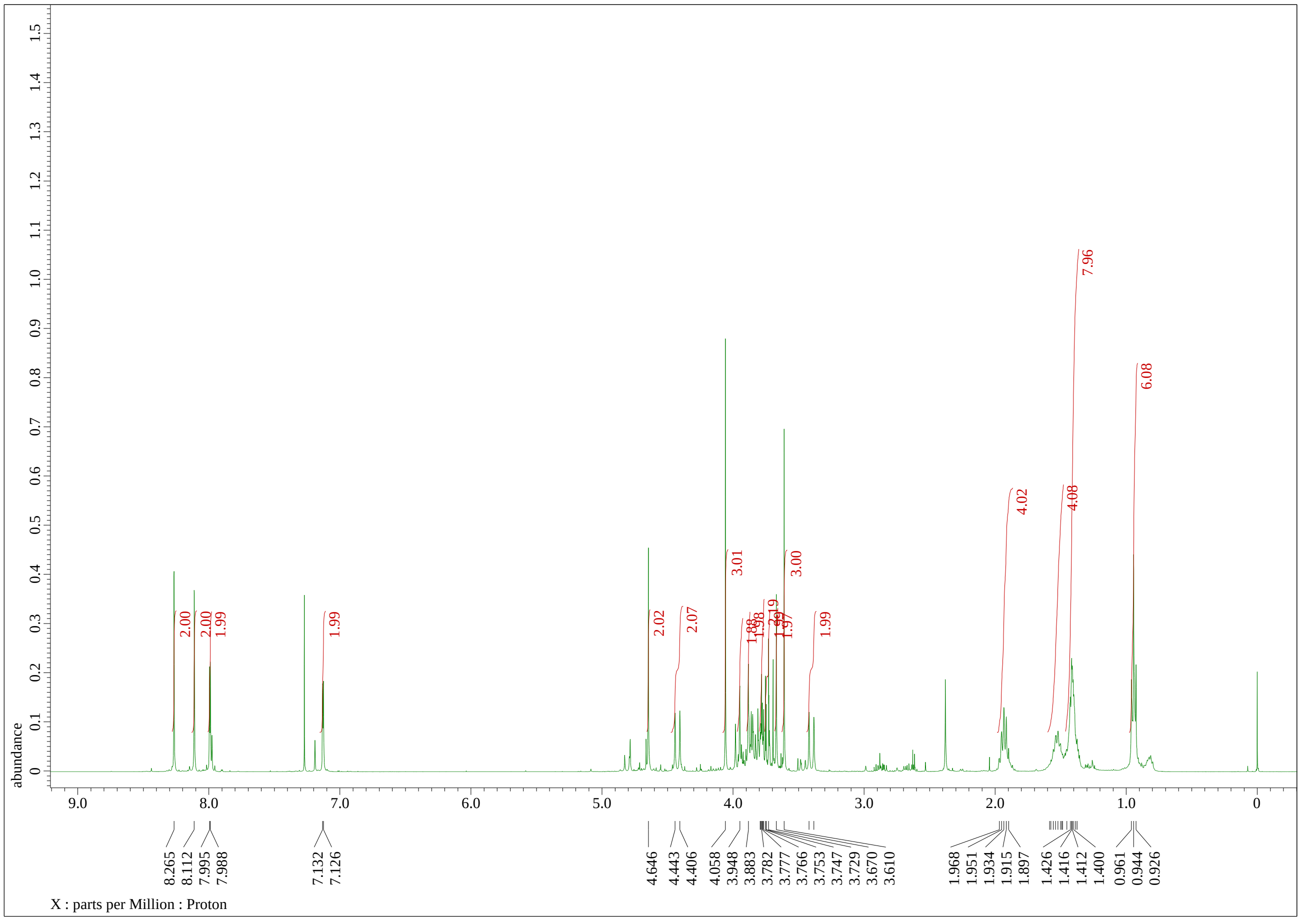
abundance
X : parts per Million : Proton

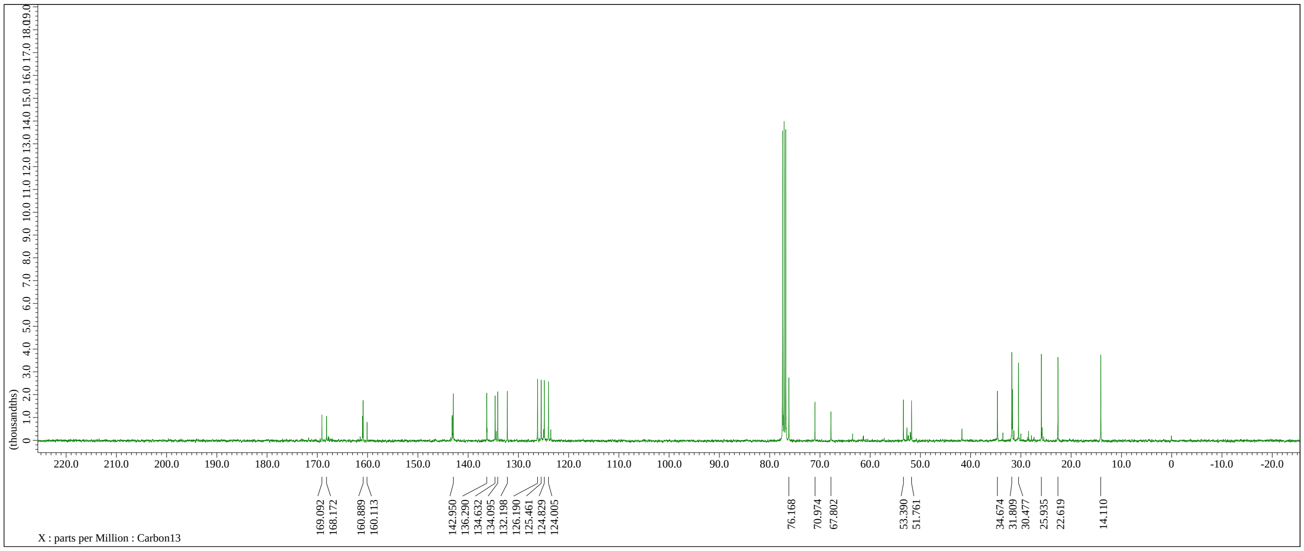
(thousandths)
X : parts per Million : Carbon13

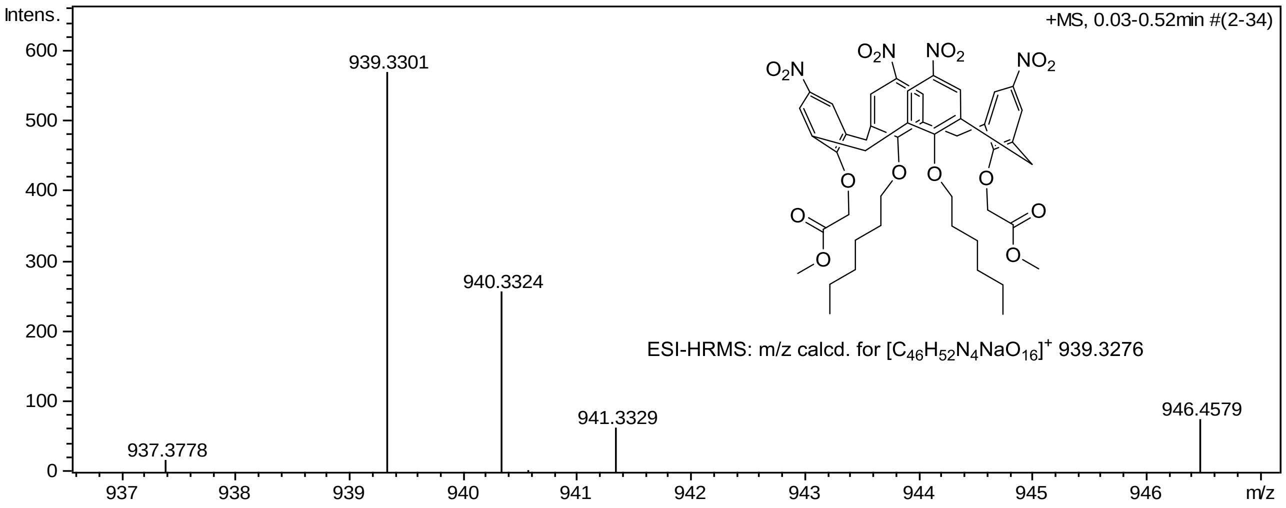

4 5,11,17,23-tetra-nitro-25,27-bis-hexyloxy-26,28-bis-[N-(2-hydroxyethyl) amino carbonyl methoxyl]-calix[4]arene

^{1}H NMR: (DMSO-d_6, 400 MHz) δ 8.33 (s, 2H), 8.21 (s, 2H), 8.04 (d, *J* = 2.8 Hz, 2H), 7.57 (t, *J* = 5.6 Hz, 1H), 7.34 (t, *J* = 5.6 Hz, 1H), 7.18 (d, *J* = 2.8 Hz, 2H), 4.69-4.62 (m, 2H), 4.42-4.36 (m, 4H), 4.13 (d, *J* = 13.6 Hz, 2H), 4.00 (s, 2H), 3.95-3.82 (m, 4H), 3.61 (d, *J* = 14.4Hz, 2H), 3.39-3.35 (m, 2H), 3.32 (t, J = 7.2 Hz, 2H), 3.25-3.21 (m, 2H), 3.17-3.13 (m, 2H), 3.02-2.97 (m, 2H), 1.87-1.80 (m, 4H), 1.45-1.40 (m, 4H), 1.36-1.31 (m, 8H), 0.89 (t, *J* = 7.6 Hz, 6H); ^{13}C NMR: ($CDCl_3$, 100 MHz) δ 168.31, 160.78, 142.71, 135.85, 134.89, 134.61, 132.62, 126.44, 125.53, 124.35, 123.56, 72.62, 71.06, 62.90, 61.71, 42.02, 34.87, 31.62, 30.25, 25.71, 22.59, 14.05; HRMS (ESI) Calcd for $C_{48}H_{58}N_6O_{16}$ ([M+Na]$^+$): 997.3807, found: 997.3816.

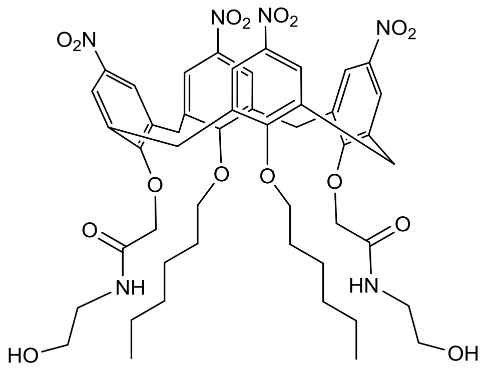

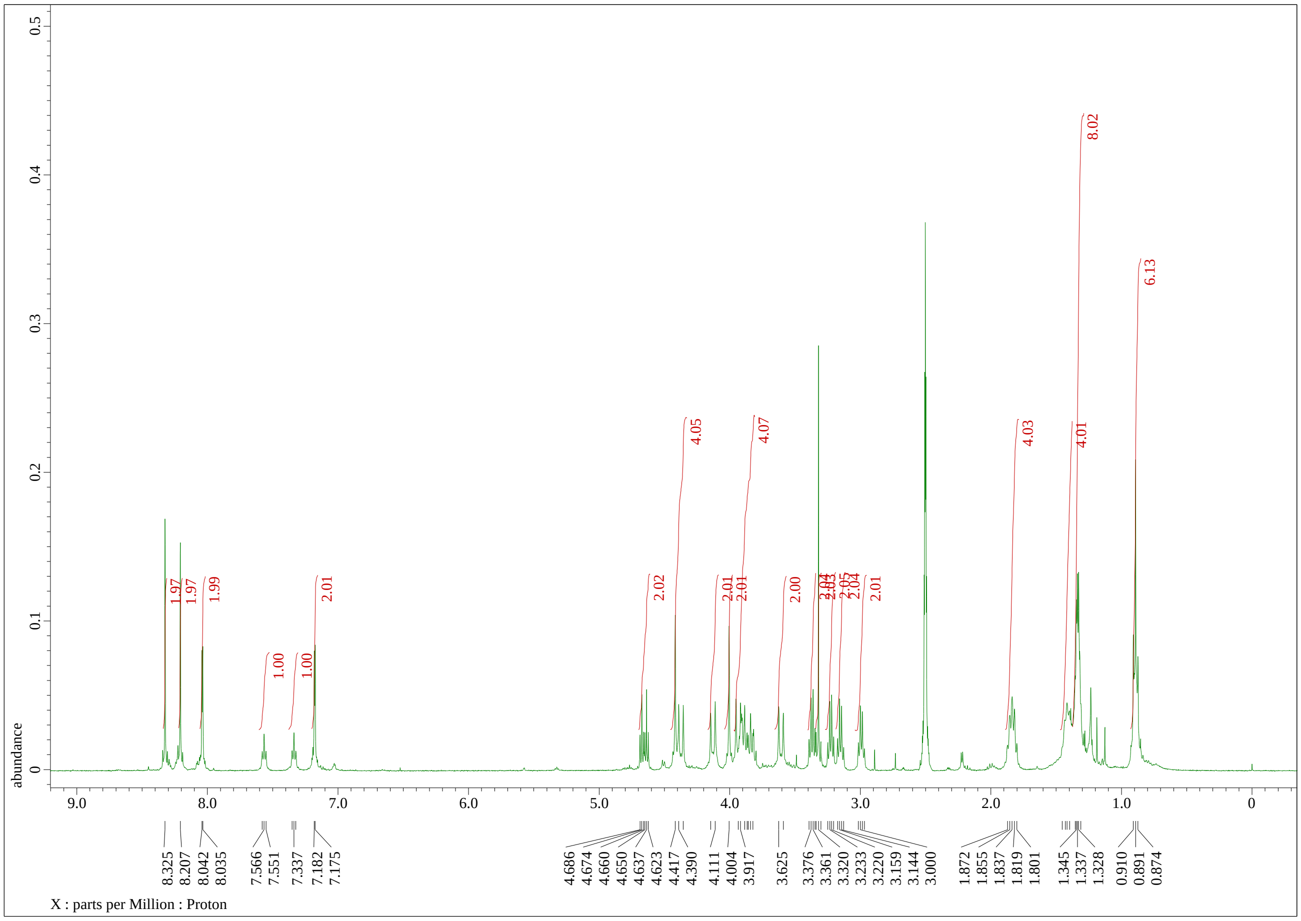
abundance
X : parts per Million : Proton

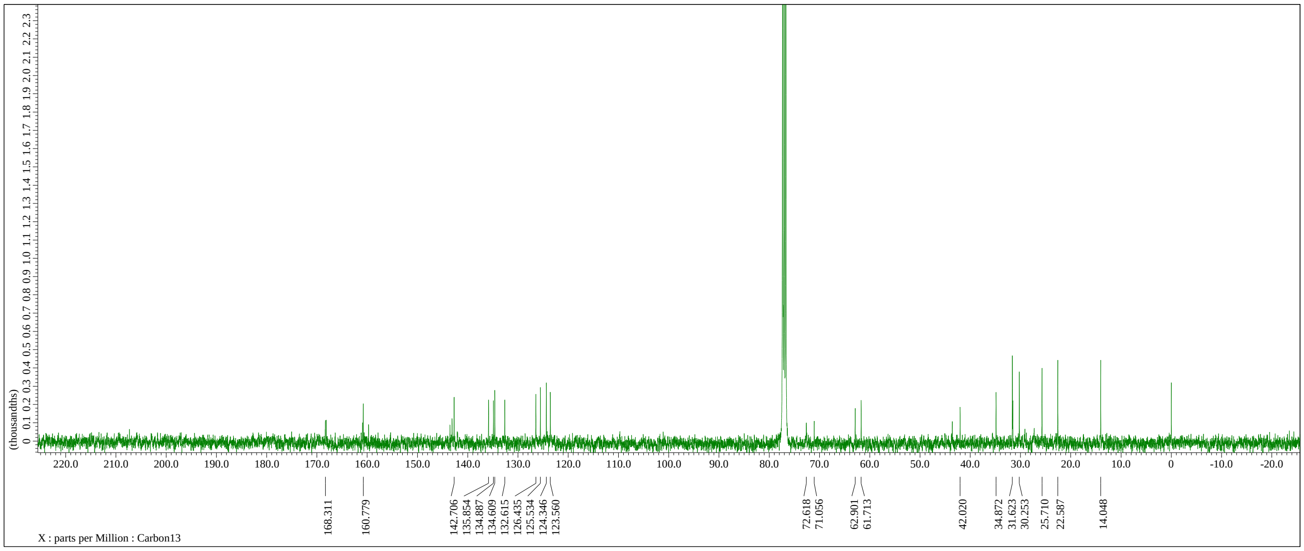
(thousandths)
X : parts per Million : Carbon13

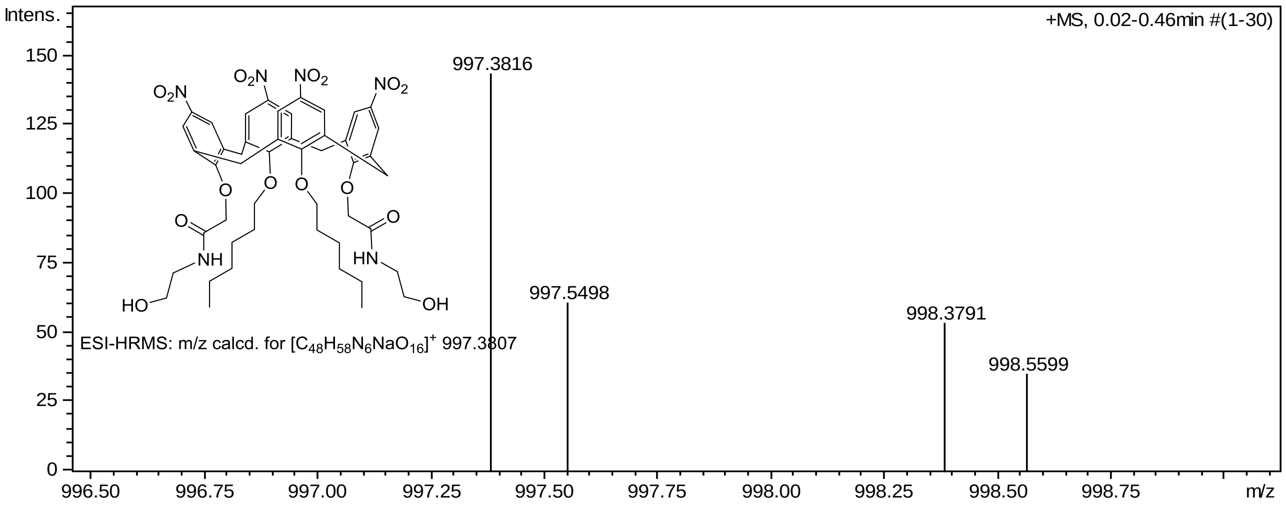

5 5,11,17,23-tetra-amino-25,27-bis-hexyloxy-26,28-bis-[N-(2-hydroxyethyl)aminocarbonyl methoxyl]-calix[4]arene

^{1}H NMR: (DMSO-d_6, 400 MHz) δ 8.41(s, 1H), 6.44 (s, 2H), 6.38 (d, J = 6.4 Hz, 1H), 6.31 (s, 2H), 6.08 (d, J = 2.0 Hz, 2H), 5.96 (d, J = 2.4 Hz, 2H), 5.39 (t, J = 5.2 Hz, 1H), 4.84 (t, J = 4.8 Hz, 1H), 4.72-4.61 (m, 8H), 4.21 (s, 4H), 3.89-3.81 (m, 4H), 3.75-3.67 (m, 4H), 3.57-3.50 (m, 4H), 3.42-3.37 (m, 4H), 3.04-2.99 (m, 2H), 2.86-2.78(m, 2H), 1.57-1.50 (m, 4H), 1.26-1.13 (m, 12H), 0.82 (t, J = 6.4 Hz, 6H) ; ^{13}C NMR: (CD$_3$OD, 100 MHz) δ 167.93, 149.28, 143.40, 137.80, 136.44, 135.49, 135.36, 117.98, 117.58, 117.46, 116.73, 75.92, 72.72, 68.45, 61.19, 42.96, 39.19, 32.98, 31.80, 30.90, 26.90, 23.80, 14.46; HRMS (ESI) Calcd for $C_{48}H_{66}N_6O_8$ ([M+Na]$^+$): 877.4840, found: 877.4852.

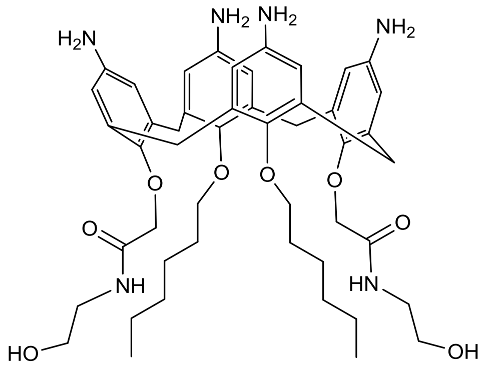

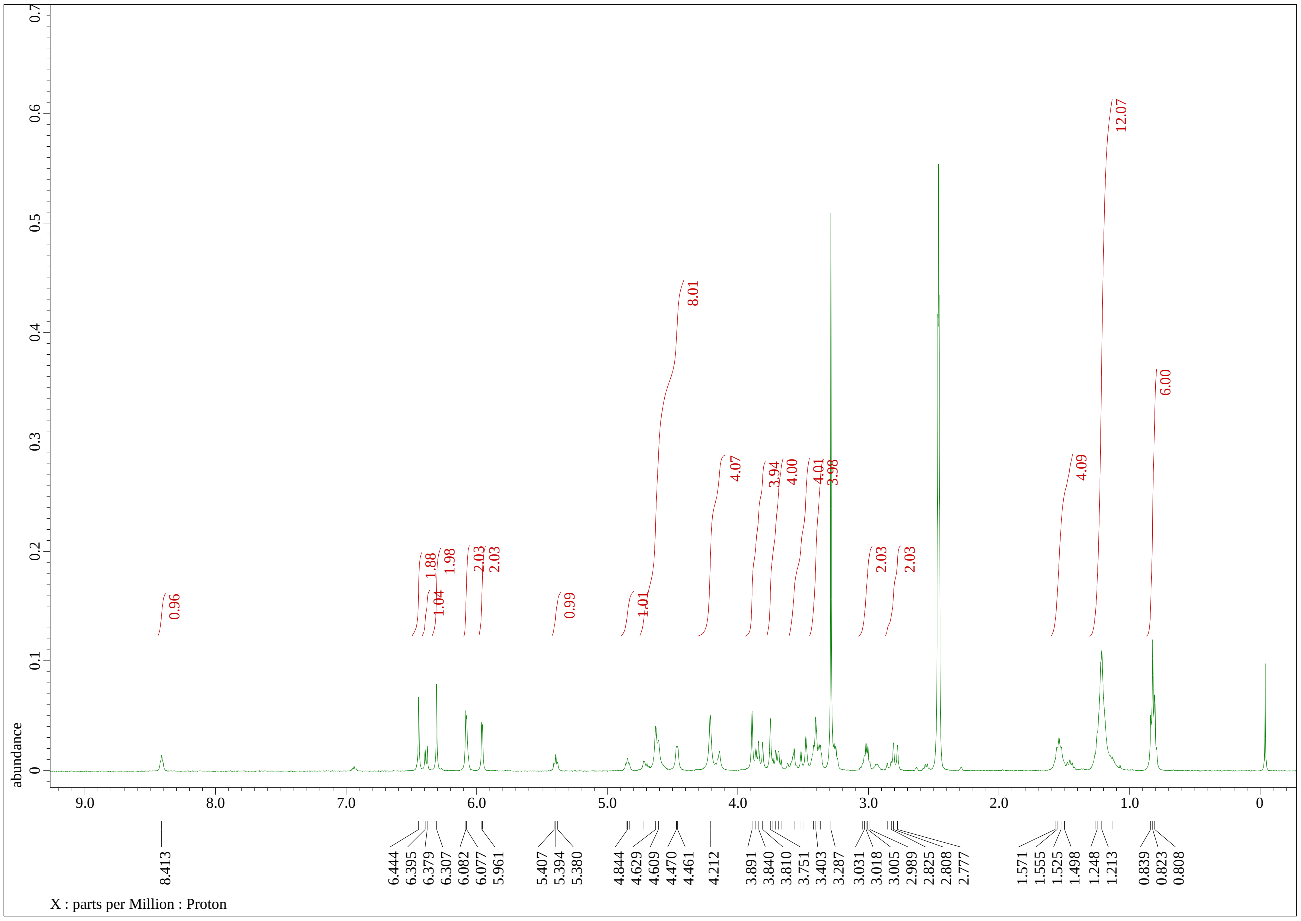
abundance
0
0.1
0.2
0.3
0.4
0.5
0.6
0.7
0.96
1.88
1.04
1.98
2.03
2.03
0.99
1.01
8.01
4.07
3.94
4.00
4.01
3.98
2.03
2.03
4.09
12.07
6.00
9.0
8.0
7.0
6.0
5.0
4.0
3.0
2.0
1.0
0
8.413
6.444
6.395
6.379
6.307
6.082
6.077
5.961
5.407
5.394
5.380
4.844
4.629
4.609
4.470
4.461
4.212
3.891
3.840
3.810
3.751
3.403
3.287
3.031
3.018
3.005
2.989
2.825
2.808
2.777
1.571
1.555
1.525
1.498
1.248
1.213
0.839
0.823
0.808
X : parts per Million : Proton

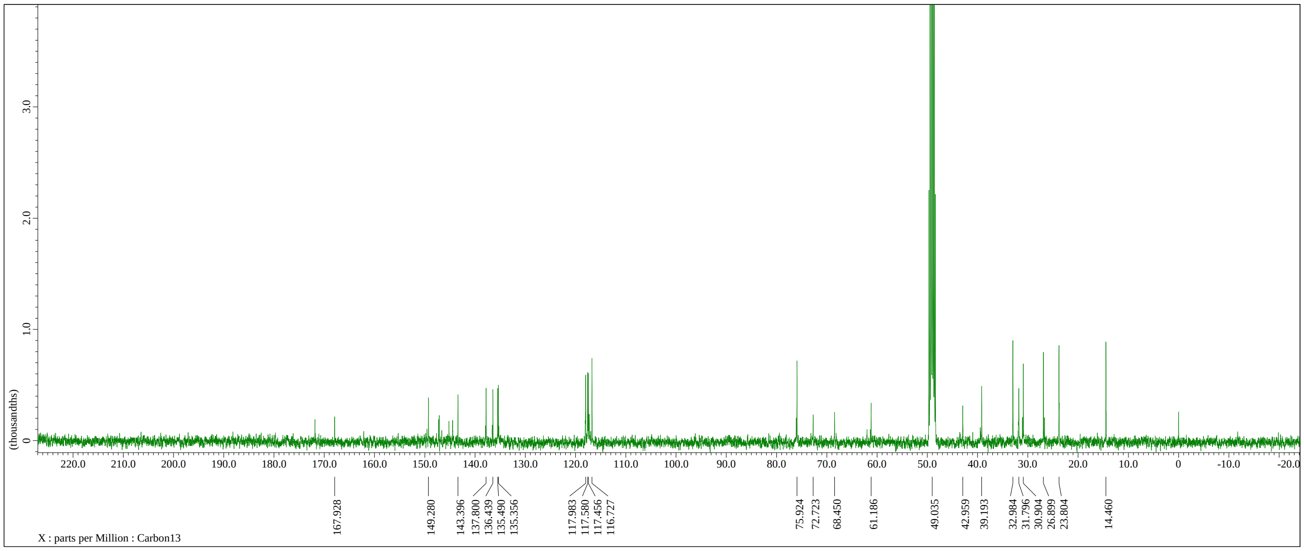
(thousandths)
0
1.0
2.0
3.0
220.0
210.0
200.0
190.0
180.0
170.0
160.0
150.0
140.0
130.0
120.0
110.0
100.0
90.0
80.0
70.0
60.0
50.0
40.0
30.0
20.0
10.0
0
-10.0
-20.0
167.928
149.280
143.396
137.800
136.439
135.490
135.356
117.983
117.580
117.456
116.727
75.924
72.723
68.450
61.186
49.035
42.959
39.193
32.984
31.796
30.904
26.899
23.804
14.460
X : parts per Million : Carbon13

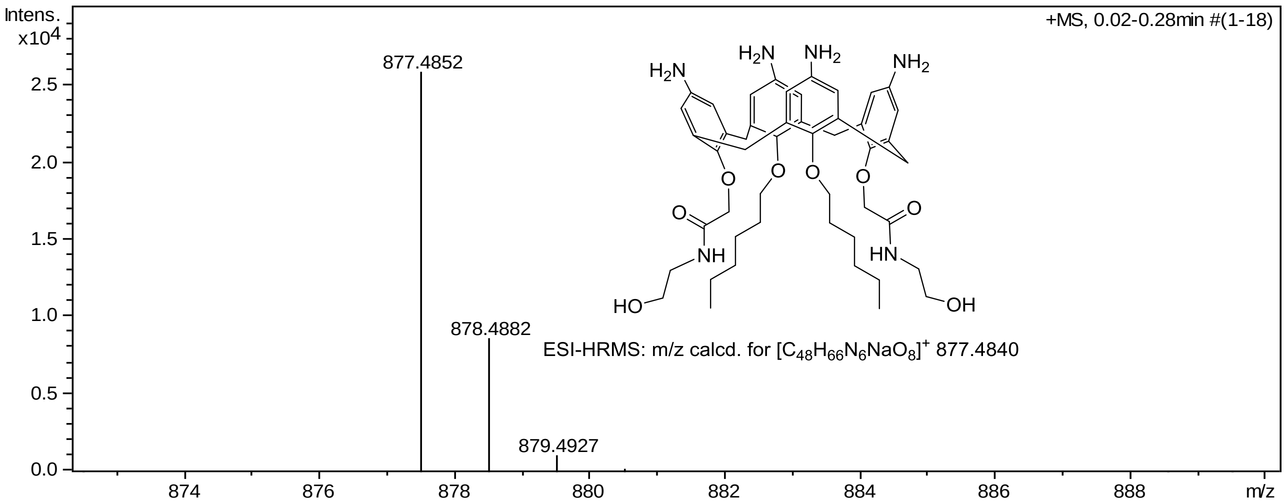

Intens.
x10^4
2.5
2.0
1.5
1.0
0.5
0.0
+MS, 0.02-0.28min #(1-18)
877.4852
878.4882
879.4927
874
876
878
880
882
884
886
888
m/z
H2N
H2N
NH2
NH2
O
O
O
O
O
O
NH
HN
HO
OH
ESI-HRMS: m/z calcd. for [C48H66N6NaO8]+ 877.4840
